# Supplementary material for: A Novel Homozygous Variant in DYSF Gene Is Associated with Autosomal Recessive Limb Girdle Muscular Dystrophy R2/2B
Source: Int J Mol Sci. 2022 Aug 11;23(16):8932. doi: 10.3390/ijms23168932 (PMC9408934; doi:10.3390/ijms23168932)
Supplement: Supplementary file 1 [file ijms-23-08932-s001.zip › ijms-1822093-supplementary.pdf]

Applied Biosystems

S/N G:573 A:375 T:682 C:722
KB.bcp
KB 1.2 Cap:14

G08_CAPN3-27-DYSF(EX45F2)
CAPN3-27-DYSF(EX45F2)
KB_3130_POP7_BDTv1.mob
Pts 2468 to 5300 Pk1 Loc:2468
Version 5.2 Patch2 HiSQV Bases: 182

Inst Model/Name 3100/AB3130xl-18232-002
Dec 11,2018 04:37PM, CET
Dec 11,2018 05:01PM, CET
Spacing:14.03 Pts/Panel800
Plate Name: PATTY-11.12.18

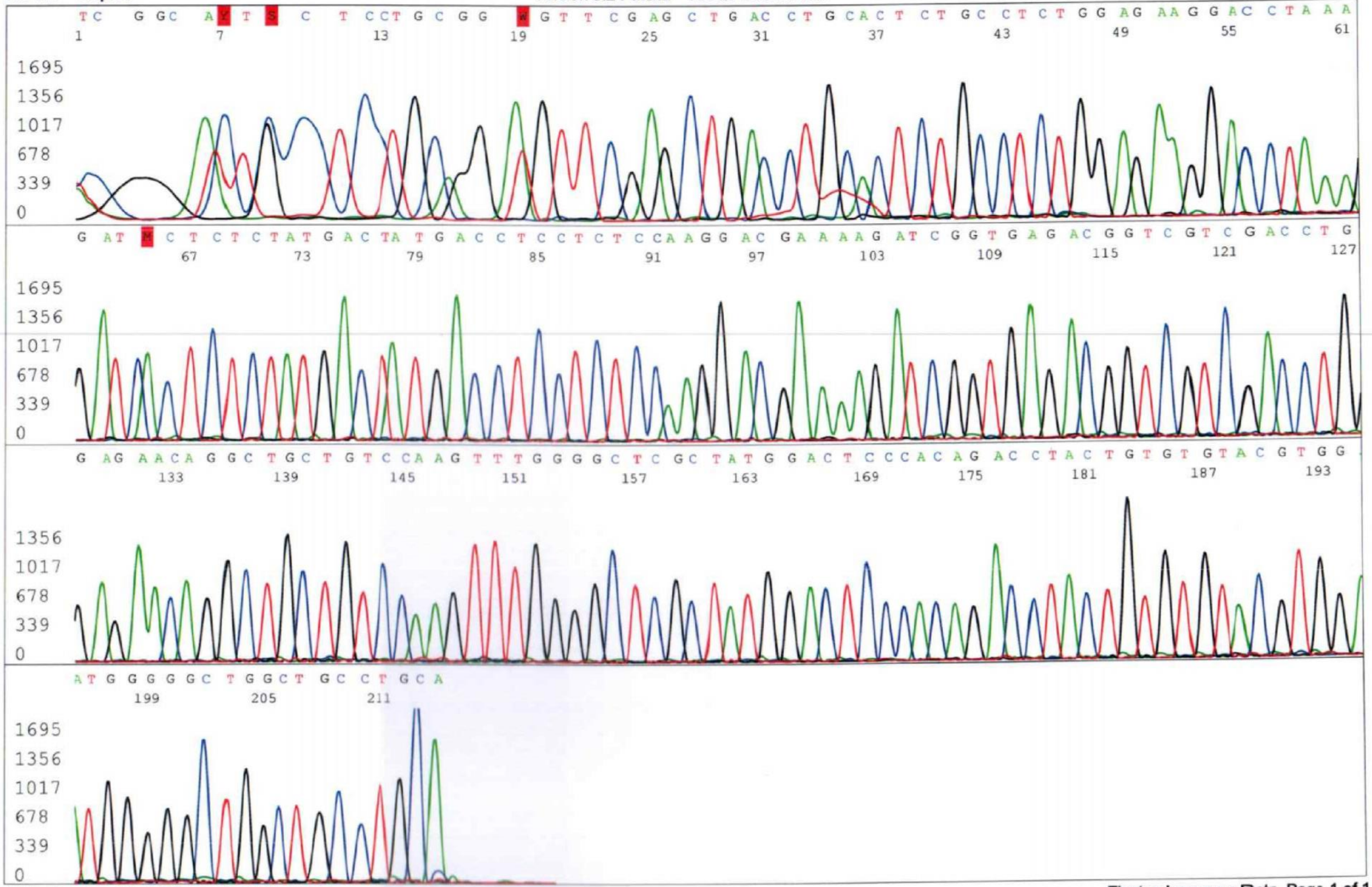

Applied Biosystems

S/N G:410 A:261 T:347 C:437
KB.bcp
KB 1.2 Cap:16

H08_CAPN3-27-DYSF(EX45R2)
CAPN-27-DYSF(EX45F2)
KB_3130_POP7_BDTv1.mob
Pts 2417 to 5300 Pk1 Loc:2417
Version 5.2 Patch2 HiSQV Bases: 181

Inst Model/Name 3100/AB3130xl-18232-002
Dec 11,2018 04:37PM, CET
Dec 11,2018 05:01PM, CET
Spacing:13.93 Pts/Panel800
Plate Name: PATTY-11.12.18

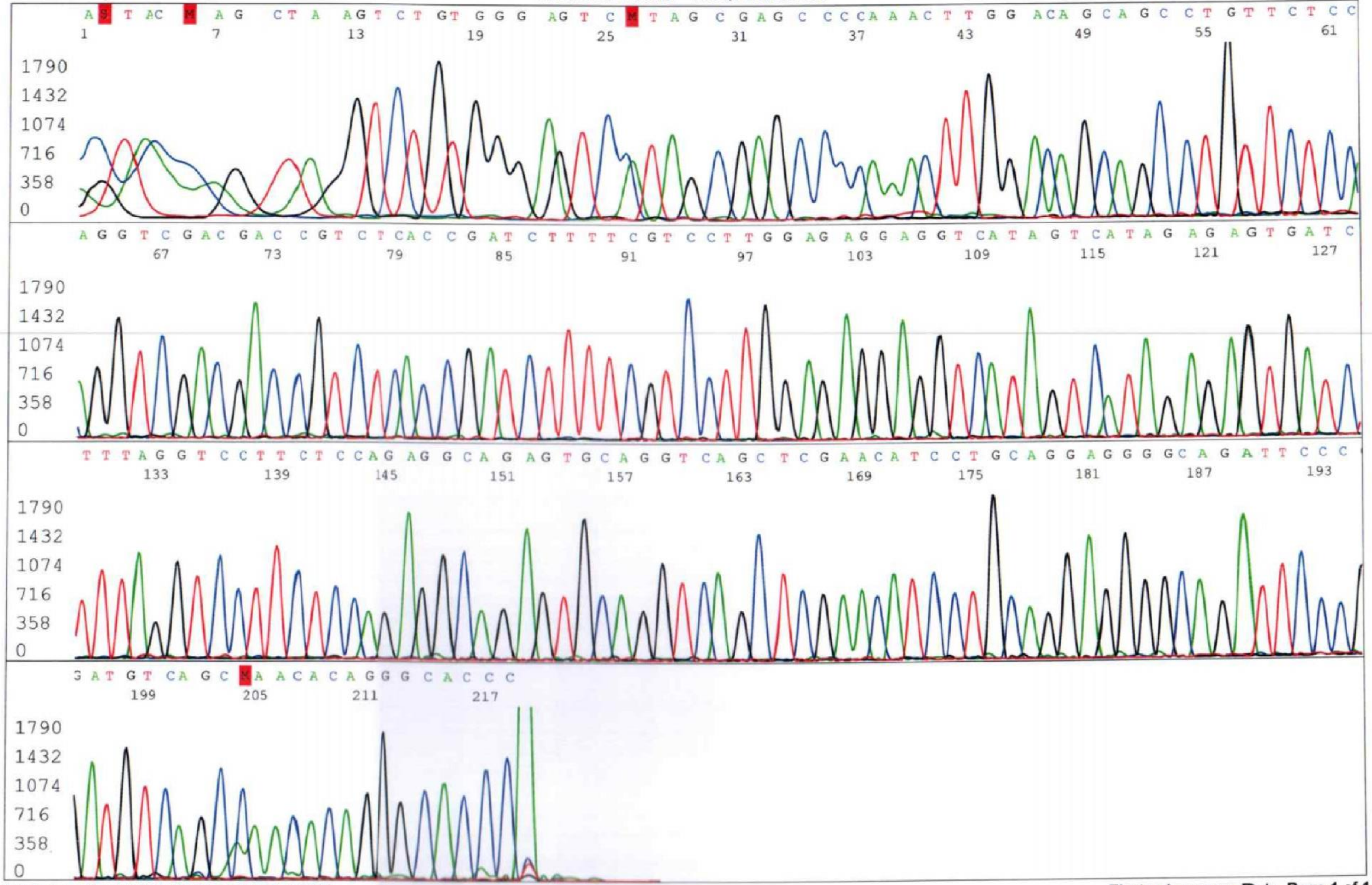


Printed on: Wed Jul 27,2022 11:07AM, CEST

Applied Biosystems

S/N G:268 A:191 T:251 C:323
KB.bcp
KB 1.2 Cap:1

A09_CAV37-DYSF(EX45F2)
CAV37-DYSF(EX45F2)
KB_3130_POP7_BDTv1.mob
Pts 2451 to 5300 Pk1 Loc:2451
Version 5.2 Patch2 HiSQV Bases: 186

Inst Model/Name 3100/AB3130xl-18232-002
Dec 11,2018 05:48PM, CET
Dec 11,2018 06:02PM, CET
Spacing:14.31 Pts/Panel800
Plate Name: PATTY-11.12.18

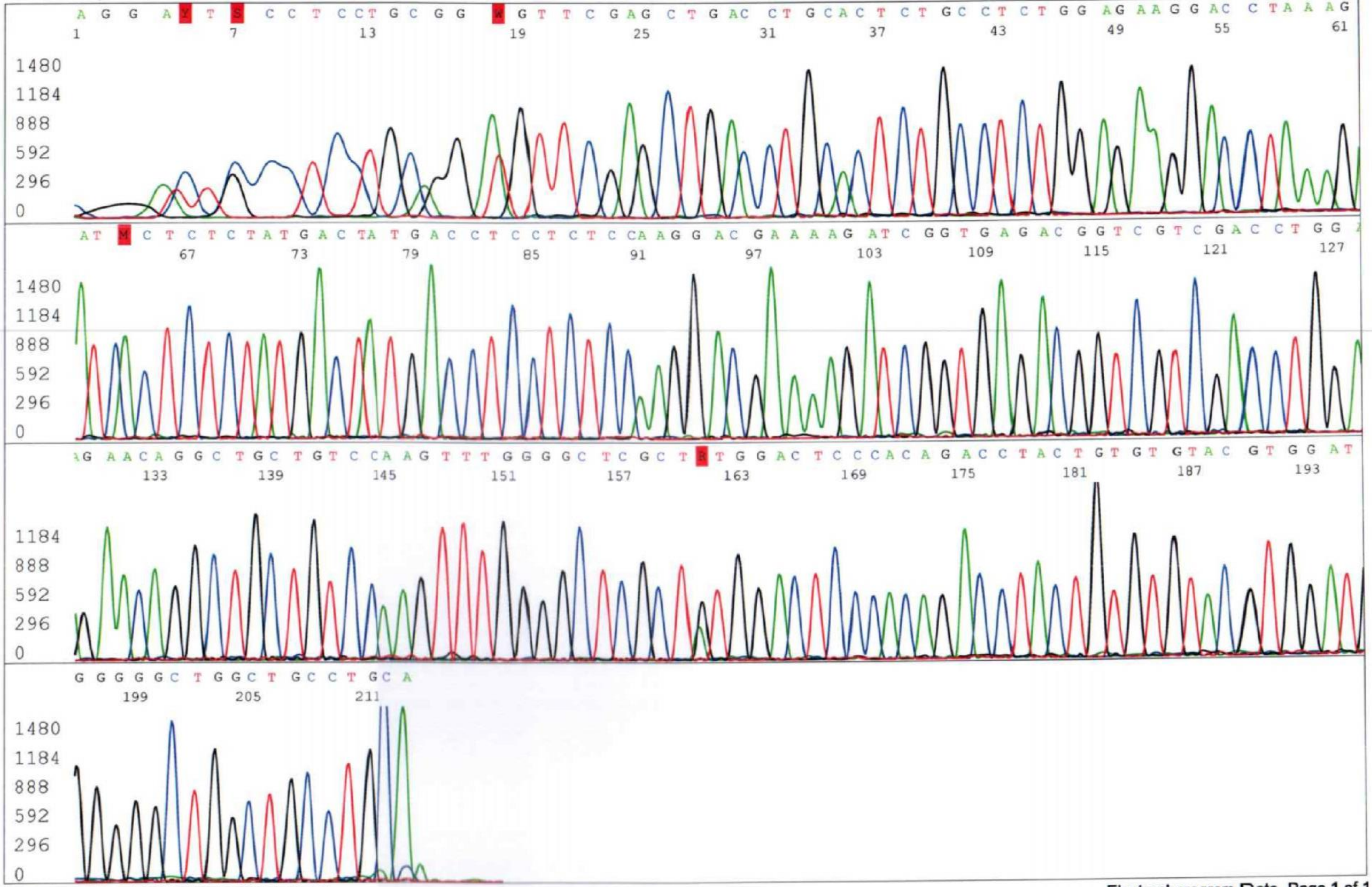

Applied Biosystems

S/N G:111 A:92 T:88 C:133
KB.bcp
KB 1.2 Cap:3

B09_CAV37-DYSF(EX45R2)
CAV37-DYSF(EX45R2)
KB_3130_POP7_BDTv1.mob
Pts 2413 to 5300 Pk1 Loc:2413
Version 5.2 Patch2 HiSQV Bases: 188

Inst Model/Name 3100/AB3130xl-18232-002
Dec 11,2018 05:48PM, CET
Dec 11,2018 06:02PM, CET
Spacing:14.16 Pts/Panel800
Plate Name: PATTY-11.12.18

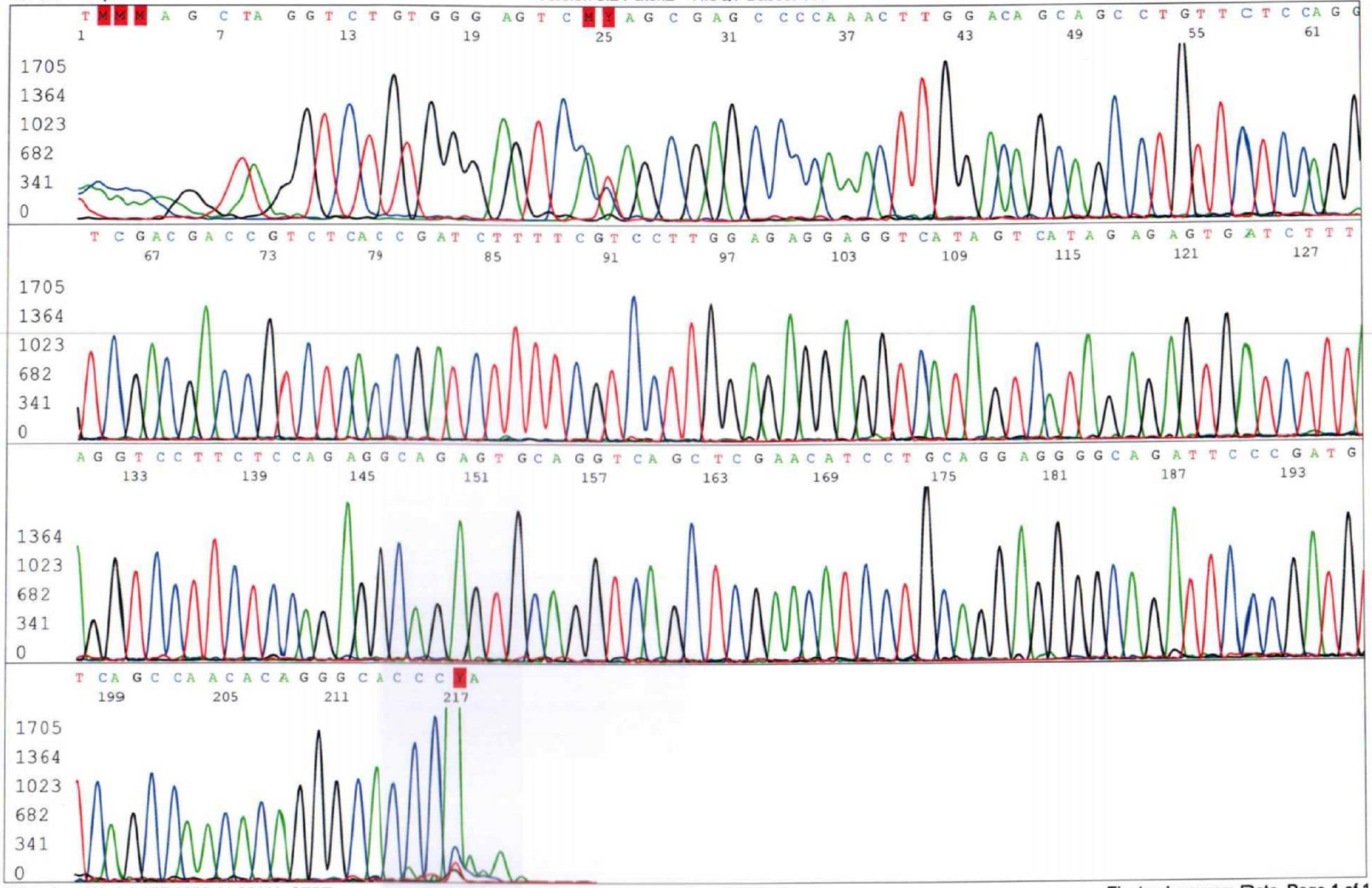


Printed on: Wed Jul 27,2022 11:09AM, CEST

Applied Biosystems

S/N G:296 A:190 T:293 C:322
KB.bcp
KB 1.2 Cap:5

C09_CAV39-DYSF(EX45F2)
CAV39-DYSF(EX45F2)
KB_3130_POP7_BDTv1.mob
Pts 2458 to 5400 Pk1 Loc:2458
Version 5.2 Patch2 HiSQV Bases: 185

Inst Model/Name 3100/AB3130xl-18232-002
Dec 11,2018 05:48PM, CET
Dec 11,2018 06:02PM, CET
Spacing:14.27 Pts/Panel800
Plate Name: PATTY-11.12.18

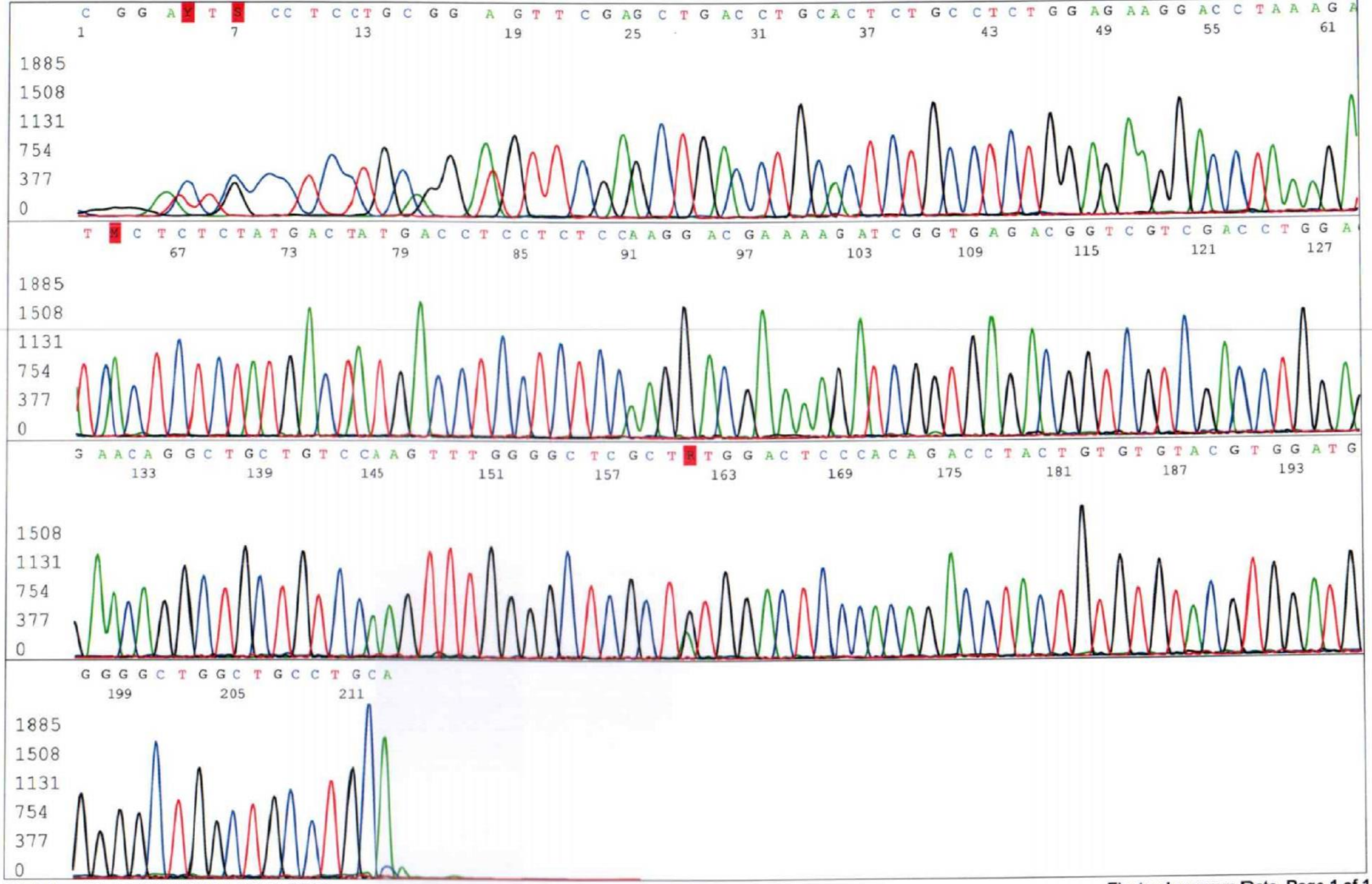

Applied Biosystems

S/N G:118 A:94 T:88 C:143
KB.bcp
KB 1.2 Cap:7

D09_CAV39-DYSF(EX45R2)
CAV39-DYSF(EX45R2)
KB_3130_POP7_BDTv1.mob
Pts 2438 to 5300 Pk1 Loc:2438
Version 5.2 Patch2 HiSQV Bases: 181

Inst Model/Name 3100/AB3130xl-18232-002
Dec 11,2018 05:48PM, CET
Dec 11,2018 06:02PM, CET
Spacing:14.19 Pts/Panel800
Plate Name: PATTY-11.12.18

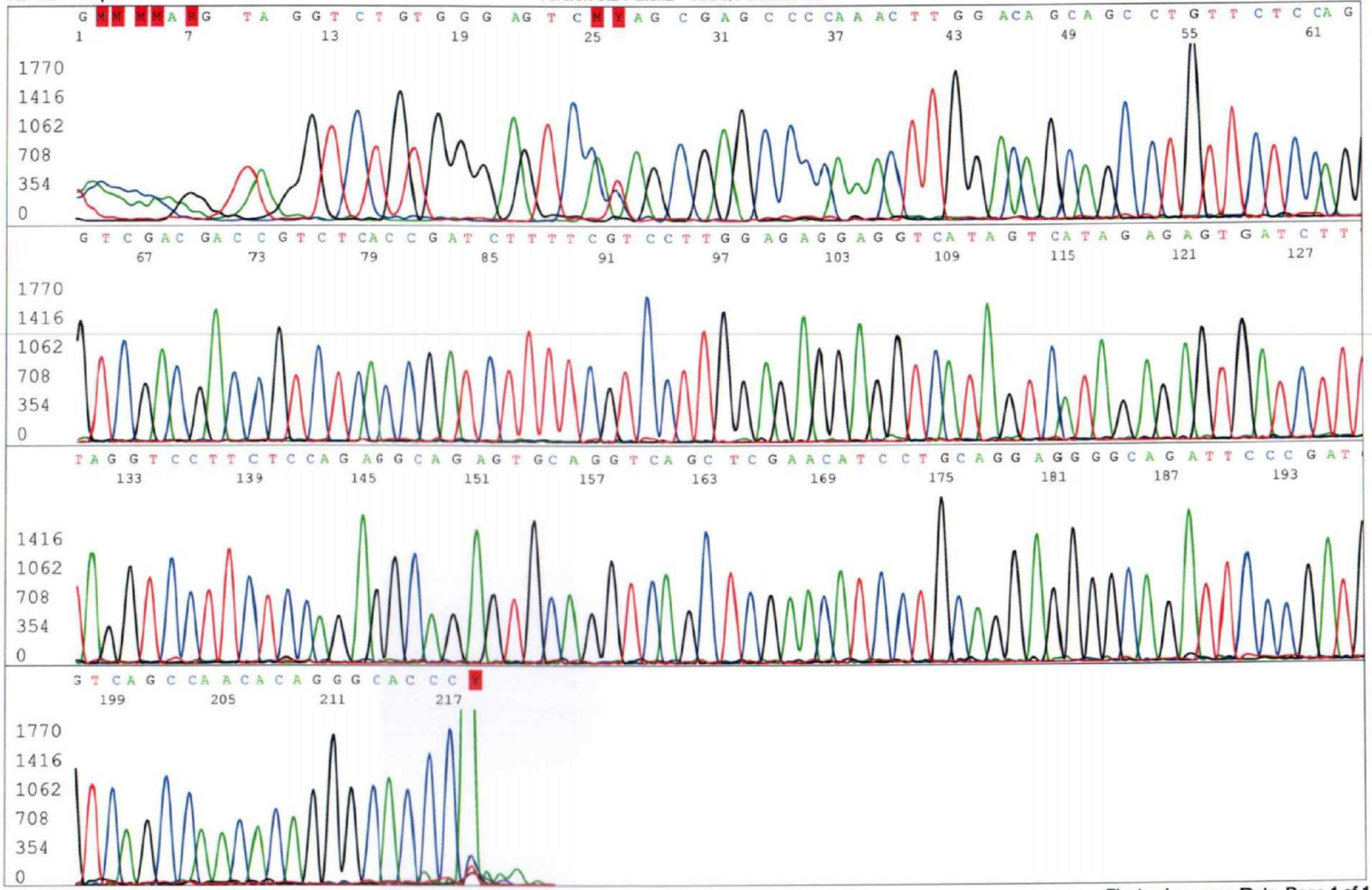

Applied Biosystems

S/N G:551 A:322 T:387 C:507
KB.bcp
KB 1.2 Cap:9

E09_CAV38-DYSF(EX45F2)
CAV38-DYSF(EX45F2)
KB_3130_POP7_BDTv1.mob
Pts 2457 to 5300 Pk1 Loc:2457
Version 5.2 Patch2 HiSQV Bases: 185

Inst Model/Name 3100/AB3130xl-18232-002
Dec 11,2018 05:48PM, CET
Dec 11,2018 06:02PM, CET
Spacing:14.28 Pts/Panel800
Plate Name: PATTY-11.12.18

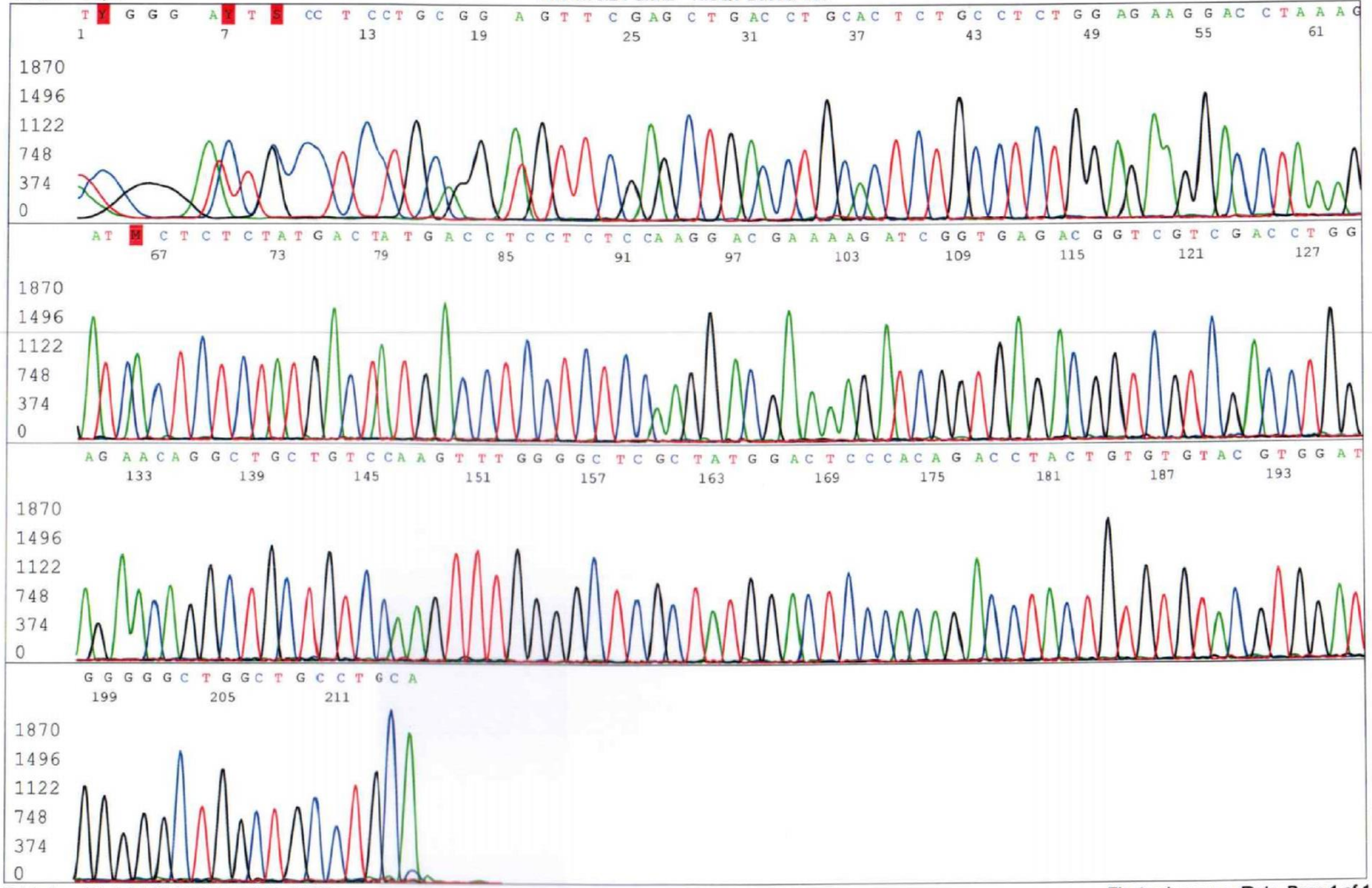


Printed on: Wed Jul 27,2022 11:03AM, CEST

Electropherogram Data Page 1 of 1

S/N G:415 A:328 T:310 C:490
KB.bcp
KB 1.2 Cap:11

F09_CAV38-DYSF(EX45R2)
CAV38-DYSF(EX45R2)
KB_3130_POP7_BDTv1.mob
Pts 2393 to 5300 Pk1 Loc:2393
Version 5.2 Patch2 HiSQV Bases: 185

Inst Model/Name 3100/AB3130xl-18232-002
Dec 11,2018 05:48PM, CET
Dec 11,2018 06:02PM, CET
Spacing:14.08 Pts/Panel800
Plate Name: PATTY-11.12.18

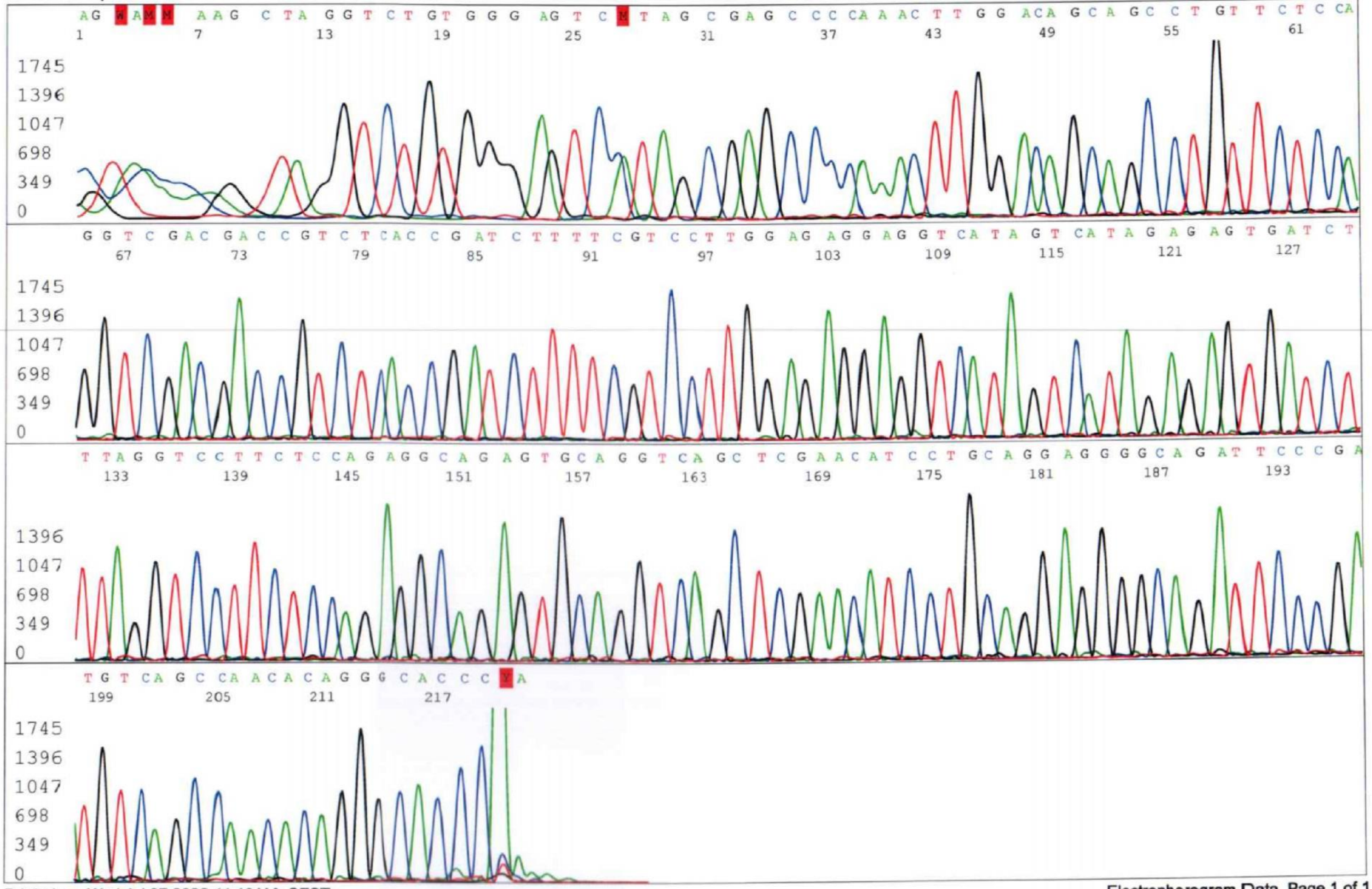


Printed on: Wed Jul 27,2022 11:10AM, CEST

Applied Biosystems

S/N G:254 A:151 T:186 C:280
KB.bcp
KB 1.2 Cap:13

G09_CAV41-DYSF(EX45F2)
CAV41-DYSF(EX45F2)
KB_3130_POP7_BDTv1.mob
Pts 2455 to 5300 Pk1 Loc:2455
Version 5.2 Patch2 HiSQV Bases: 180

Inst Model/Name 3100/AB3130xl-18232-002
Dec 11,2018 05:48PM, CET
Dec 11,2018 06:02PM, CET
Spacing:14.08 Pts/Panel800
Plate Name: PATTY-11.12.18

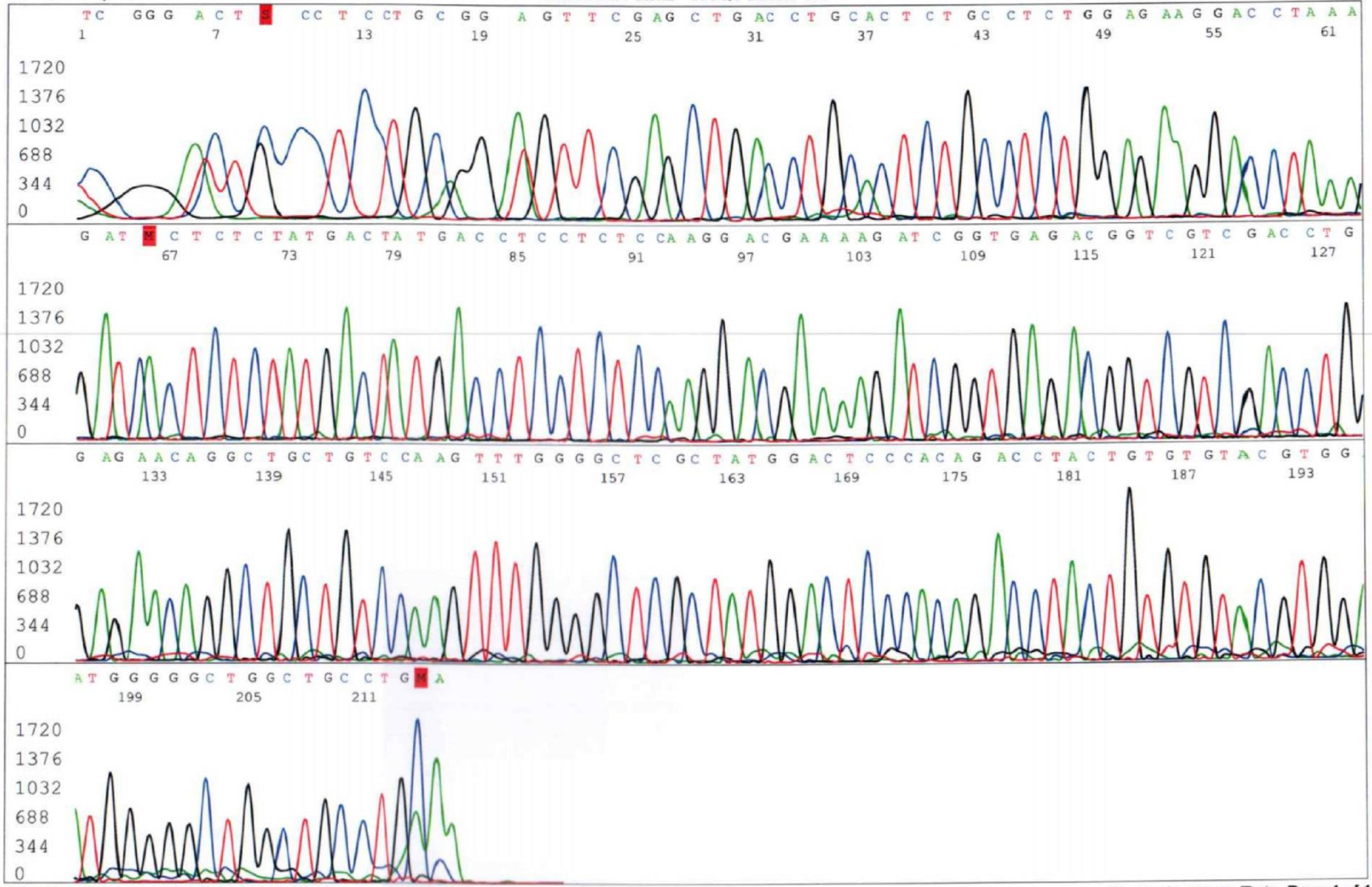


Printed on: Wed Jul 27,2022 11:11AM, CEST

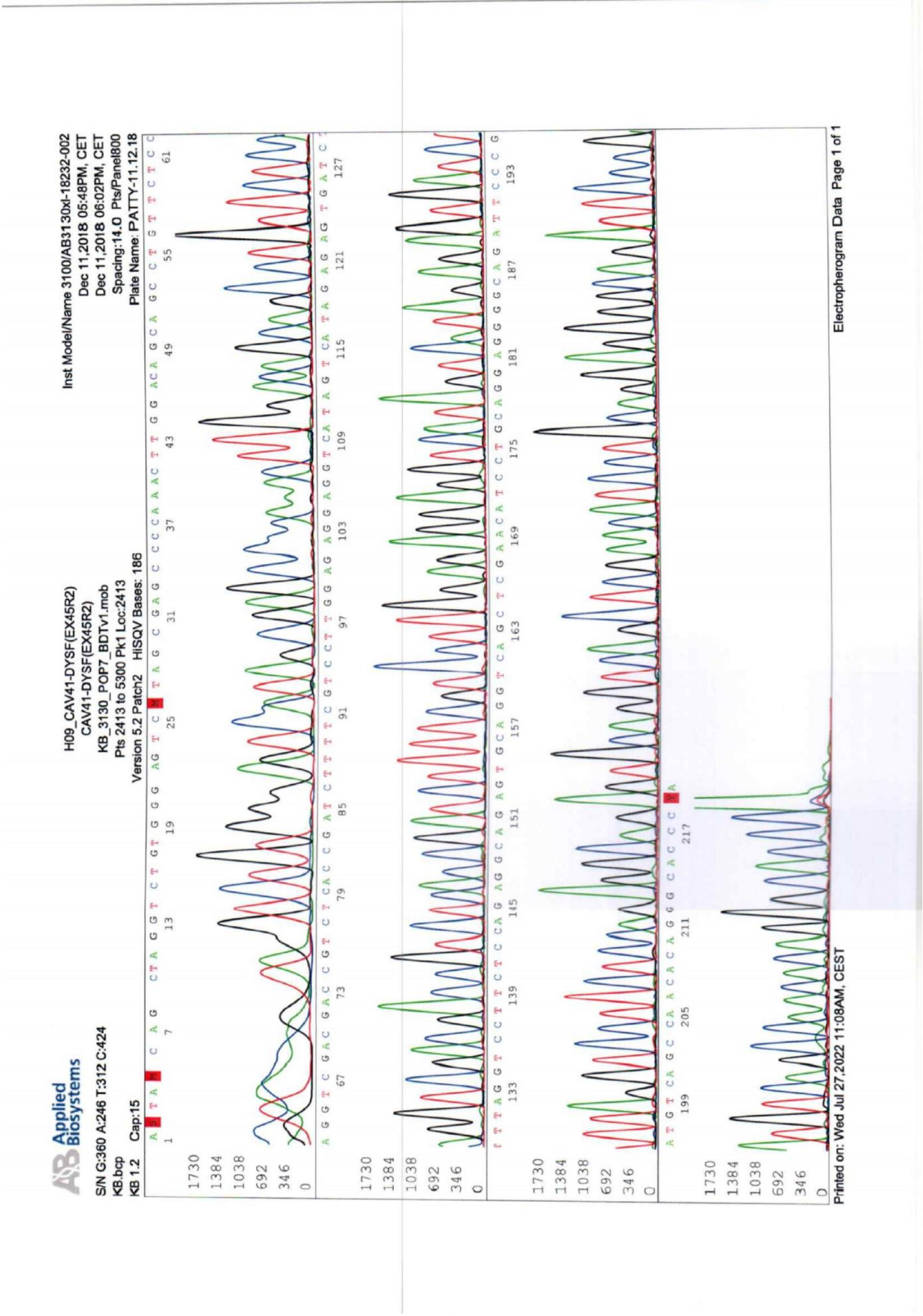


Chromatograms related to the five subjects of the studied family, obtained by Sanger direct sequencing with Forward and Reverse primers. The sequences are all related to exon 45 of the DYSF

gene showing the presence in heterozygosity or in homozygosity of the missense variant c.5033G>A, p.Cys1678Tyr.
